# Live birth rates after resolution of endometrial cavity fluid in frozen embryo transfer cycles

**DOI:** 10.1186/s12958-023-01149-8

**Published:** 2023-10-27

**Authors:** Vincent Nguyen, Aaron Jackson, Jenna Gale

**Affiliations:** 1 Dept of Obstetrics and Gynecology, University of Ottawa, 501 Smyth Road, Ottawa, ON K1H 8M5 Canada; 2 Ottawa Fertility Centre, Green Valley Crescent, Ottawa, ON 100-955, K2C 3V4 Canada

**Keywords:** Endometrial cavity fluid, Fluid, FET, Infertility

## Abstract

**Research question:**

Are live birth rates affected in frozen embryo transfer cycles that develop transient endometrial cavity fluid that resolves by day of embryo transfer?

**Design:**

The first frozen blastocyst transfer cycle between January 1st, 2016 and December 31st, 2019 were included in this retrospective cohort study at an academic fertility center. The presence or absence of endometrial cavity fluid (ECF) detected on initial ultrasound and at time of transfer was recorded. Patients who had persistent ECF at time of transfer were excluded from the study. The primary outcome was live birth rate in the group with resolved ECF relative to the group without ECF.

**Results:**

A total of 1034 frozen blastocyst transfer cycles were included, 54 with resolved ECF and 980 without ECF. Adjusted analyses were performed using a log-binomial regression model. Live birth rates were 35.2% and 34.2%, adjusted risk ratio 1.00 [95% CI 0.70-1.50] in the two groups, respectively.

**Conclusion:**

Live birth rates in frozen embryo transfer cycles are equivalent between patients with resolved endometrial cavity fluid compared to those who never had endometrial cavity fluid. Our findings suggest that the presence of endometrial cavity fluid is likely not detrimental to live birth rates if the fluid spontaneously resolves by the time of embryo transfer.

## Introduction

Endometrial cavity fluid (ECF) in the context of assisted reproductive technology (ART) describes fluid in the endometrial cavity sporadically detected during sonographic surveillance in any given cycle. The reported incidence of ECF ranges from 3.0 to 8.0% in fresh cycles [13], with one study reporting 2.8% in frozen [8]. The origins of ECF are controversial, however its composition can range from mucous, blood, tubal or endometrial secretions. ECF may commonly develop transiently after HCG injection, and has been often associated with hydrosalpinx [1, 4, 9, 13].

Regardless of the source of ECF, many retrospective studies and more recently a meta-analysis by Liu et al. demonstrate that clinical pregnancy rates are lower among women with ECF than those without [13]. The mechanism of this deleterious effect is largely unknown, however many predisposing factors such as hydrosalpinx or subclinical uterine infections may be contributory. Other theories postulate that the presence of ECF may interfere with the attachment of an embryo to the endometrial surface, or perhaps intrauterine cytokines or prostaglandins may harbour an embryotoxic milieu unsuitable for apposition [5, 17, 18].

Irrespective of its mechanism, the threat of ECF on the success of any given cycle remains unclear, thus many centres have adopted protocols to treat ECF once detected. A few case series have reported various approaches, including aspirating ECF prior to transfer [3, 7, 15] or transmyometrial embryo transfer [10, 19], although the evidence to support these practices is lacking.

Perhaps the most common approach, adopted by our centre, includes expectant management with close ultrasound surveillance to determine if ECF remains present on day of transfer. If persistent the embryo transfer would be cancelled, but if resolved then transfer may continue. While in theory the disappearance of ECF may provide a sense of security for a provider to continue with embryo transfer, there is a paucity of evidence to support this practice. Given that the origin and mechanism of ECF is controversial, we have yet to determine if its presence may have left lasting effects that may hinder the success of transfer.

We therefore sought to validate if expectant management was suitable for the management of ECF in frozen embryo transfer (FET) cycles. We present a retrospective cohort study comparing live birth rates in FET cycles between patients with transient ECF which resolved by time of embryo transfer, compared to those in whom ECF was never observed.

## Materials and methods

### Study population

Patients who started a frozen embryo transfer cycle between January 1st 2016 and December 31st 2019 at the Ottawa Fertility Centre (OFC) were eligible for inclusion. Patients were included in the analysis if they underwent FET with blastocysts cryopreserved by vitrification, created from their own oocytes or donor oocytes with either partner or donor sperm, whether embryos cryopreserved were surplus after fresh embryo transfer or were cryopreserved in a freeze-all cycle. Patients using a gestational carrier were included. Cycles employing PGT-M without PGT-A were included. Only the first FET cycle of each patient was included in the study.

Patients were excluded if a uterine anomaly, abnormal cavity (submucosal fibroid, endometrial polyp, septum), or hydrosalpinx was present at time of embryo transfer. Patients were also excluded if they used a controlled ovarian stimulation protocol rather than a true natural or programmed HRT cycle. Cycles employing PGT-A were excluded. Patients with ECF at the time of embryo transfer were excluded from the study, regardless of if the cycle was cancelled or not.

Vitrification of blastocysts occurred on day 5. Patients were identified through an in-house medical record system, and clinic linkage to the Canadian Assisted Reproductive Technologies Register (CARTR Plus) provided birth outcome data. The study protocol was reviewed by the Ottawa Health Science Network Research Ethics Board and deemed exempt from OHSN-REB review, as a quality improvement study. Data was housed on a local secure server and analysis available only to study authors.

All patients undergoing FET had an initial ultrasound to assess endometrial thickness, at which point ECF would be documented if present. Patients undergoing a Natural Cycle (NC) protocol, described below, had their initial ultrasound the day of LH surge or the day after, as determined via serum LH of ≥ 30nmol/L. Patients undergoing a Hormone Replacement (HR) FET protocol, described below, had their initial ultrasound performed after 2–3 weeks of vaginal estrogen. If ECF was detected at an initial scan, a follow-up ultrasound was performed on the day prior to embryo transfer to ensure resolution. If ECF persisted, the cycle would typically be cancelled unless the patient wished to continue with FET despite persistent ECF. If ECF resolved, then FET would continue as scheduled.

Blastocysts were graded based on Gardner’s scoring system [6]. At our centre, only good and best quality blastocysts (B1-3 and greater) were selected for cryopreservation, unless exceptional circumstances prevailed. The number of embryos transferred in the cycle was at the discretion of the physician in discussion with the patient, and was pre-determined at a follow-up appointment prior to the FET cycle. The decision to transfer 1 vs. 2 embryos was made with the patient by taking in to account the patient age, number of prior embryo transfers and patient factors posing additional risk in pregnancy given a multiple gestation, with a tendency at our clinic toward elective single embryo transfer.

### Natural cycle FET protocol

The “true NC” approach was employed at our centre throughout this study period, whereby ovulation occurs spontaneously and was not triggered with exogenous hormones. Women were considered candidates for NC protocol if they had regular menstrual cycles, ranging in length between 27 and 32 days, a mid luteal phase serum progesterone ≥ 30 nmol/L typically measured 6–8 days post urinary LH surge, and there was no luteal phase concern (i.e. significant luteal phase spotting, or evidence of a short luteal phase). The protocol involved daily serial morning bloodwork sampling for estradiol and LH, typically started 3–4 days prior to the expected LH surge, until the LH surge was observed. The LH surge was defined as the attainment of a serum LH ≥ 30 IU/L with a dropping estradiol, or the highest level LH ≥ 30 IU/L given that a dropping serum estradiol was not a strict criterion. The day on which this was observed was considered day 0 of the cycle, as is standard within the FET literature [14].

Once an LH of ≥ 30 IU/L was identified, a pelvic ultrasound was performed to obtain a measurement of endometrial thickness. After a documented LH of ≥ 30 IU/L and endometrial thickness ≥ 7 mm, embryo transfer was scheduled on day 4. Exogenous progesterone was not administered for luteal phase support. If a patient did not meet these criteria, the cycle was cancelled, and the patient was scheduled for follow-up with their physician to discuss either another attempt at the NC protocol or switching to an HR protocol.

### Hormone replacement FET protocol

Patients were selected for HR FET if they did not meet the criteria for NC as outlined above, or if they elected to proceed with this approach for other reasons (i.e. ease of scheduling and fewer visits for bloodwork and ultrasound). Our standard hormone replacement protocol involves estrogen priming with an escalating vaginal micronized estradiol (Estrace) administration starting on cycle day 3–5 and continued for 2–3 weeks, or vaginal micronized estradiol 2 mg three times daily for 14–18 days (estradiol administration was based on provider preference), after which transvaginal ultrasound for endometrial thickness and serum estradiol and progesterone were assessed. If patients met the requirements of endometrial lining of ≥ 7 mm, serum estradiol ≥ 650 pmol/L and progesterone < 5 nmol/L, they were advised to start progesterone in oil IM 50 mg daily. The embryo transfer was scheduled four days after progesterone start. In cases of inadequate endometrial thickness or serum estrogen, ongoing estrogen supplementation, typically for an additional week at the same or higher (in the setting of a low or low-normal serum estradiol level) doses, was employed. Endometrial thickness and serum estradiol were re-checked after additional estrogen and if adequate, progesterone was commenced, and FET scheduled. If inadequate, the cycle was either cancelled, or the patient could elect to proceed with progesterone and scheduling of FET after a discussion with the physician. Estrogen and progesterone supplementation were then continued until either a negative serum pregnancy test or until 10 weeks’ gestational age.

### Outcome assessment

The primary outcome was live birth after FET. A live birth was defined as an infant born showing any signs of life, or at least ≥ 20 weeks’ gestational age or weighing 500g. Secondary outcomes included rate of positive serum human chorionic gonadotropin (hCG), clinical intrauterine pregnancy, miscarriage, ectopic and stillbirth pregnancy. Serum hCG was measured approximately 12–14 days after FET, and measurements ≥ 5 IU/L were considered positive. Clinical intrauterine pregnancy was defined as the presence of a gestational sac and yolk sac on transvaginal ultrasound. Miscarriage was defined as a birth outcome where a clinical pregnancy was diagnosed but no fetus development could be seen at < 20 weeks’ gestation. Stillbirth was defined as a pregnancy loss at ≥ 20 weeks’ gestation.

### Statistical analysis

Patient and cycle characteristics were described using frequencies and proportions for categorical variables and statistical comparison were done with Fisher Exact test for non-parametric data and Chi square for parametric data. We described normal continuous variables using means and standard deviations and compared groups using a two-sided t-test. We fit a multivariable log-binomial regression model with a priori variables for the primary and secondary outcomes, adjusting for patient age at oocyte retrieval, body mass index, PCOS or other ovulatory disorder, tubal factor and endometriosis as indication for treatment, use of donor oocytes, and the number of blastocysts transferred. Overall live birth, positive hCG, clinical intrauterine pregnancy, miscarriage, ectopic and stillbirth pregnancy rates were compared between the two groups. Adjusted risk ratios with 95% confidence intervals were performed.

To detect an absolute difference of 25% in live birth rate between the two groups, a sample size of 40 patients was required in the study group. Statistical analyses were performed using SAS statistical software version 9.4 (SAS Institute Inc., Cary, NC).

## Results

A total of 1711 frozen embryo transfer cycles were eligible for inclusion in this study. Of these, 1083 were the first cycle within the time period of the study. Ten patients were excluded related to uterine anomaly at the time of embryo transfer (two unicornuate uterus, six had polyp and one had a submucosal fibroid present at time of FET, one patient had a non-resected partial septum). Two patients were excluded because there was a hydrosalpinx present at time of embryo transfer. Five patients were excluded who had fluid at their initial check, given that we did not have information about whether fluid was present at the time of embryo transfer. Five patients were excluded because a controlled ovarian stimulation was utilized for frozen embryo transfer protocol. Cycles utilizing PGT-A were excluded from the analysis (27 cycles).

Five patients opted to continue with FET despite persistent fluid in the cavity and were excluded from the study. Of these cases, two did not achieve clinical IUP with one patient having had the ECF aspirated prior to transfer. One patient with persistent fluid also had concurrent hydrosalpinx which resulted in clinical IUP but ultimately ended in miscarriage. One patient developed an ectopic pregnancy. One patient achieved a live birth.

After application of exclusion criteria, 1034 cycles were included in the final analysis. Of these, transient ECF which resolved spontaneously was detected in 54 cycles, with an incidence of 5.2% (54/1034). Table 1 outlines the baseline characteristics of study participants. No patients in either group had history of a prior abdominal myomectomy. Table 2 outlines cycle specific characteristics of the FET cycle included in this analysis. Pre-freeze embryo quality was missing for 17 study participants (1 within the study and 16 within the control group). Table 3 outlines primary and secondary outcomes. Live birth rates were 35.2% among women with resolved ECF and 34.2% among those without ECF, adjusted risk ratio 1.0 [95% CI 0.7–1.5].

**Table 1:** Baseline characteristics of study participants

Variable	Fluid resolved (N = 54)	No fluid (N = 980)
Age at time of egg retrieval	33.2 (5.1)	33.4 (4.0)
Age at time of embryo transfer	36.0 (5.5)	35.1 (4.3)
BMI	25.3 (5.0)	25.0 (4.8)
Number of prior pregnancies	0.9 (1.0)	1.1 (1.3)
Number of prior pregnancies		
0 1 2 3 >=4	20 (37.0) 26 (48.2) 4 (7.4) 3 (5.6) 1 (1.9)	396 (40.4) 332 (33.9) 143 (14.6) 64 (6.5) 45 (4.6)
Number of prior deliveries	0.5 (0.6)	0.4 (0.7)
Prior operative hysteroscopy Polypectomy Myomectomy Adhesiolysis Septoplasty	8 (14.8) 2 (3.7) 3 (5.6) 2 (3.7) 1 (1.9)	103 (10.5) 74 (7.6) 9 (0.9) 17 (1.7) 8 (0.8)
Fertility diagnosis		
Male factor Tubal factor Endometriosis PCOS Other ovulatory dysfunction Uterine factor Unexplained	25 (46.3) 6 (11.1) 3 (5.6) 11 (20.4) 3 (5.6) 2 (3.7) 6 (11.1)	573 (58.5) 104 (10.6) 63 (6.4) 100 (10.2) 42 (4.3) 11 (1.1) 103 (10.5)
Number of prior IVF cycles		
0 1 2 >=3	3 (5.6) 30 (55.6) 14 (25.9) 7 (13.0)	18 (1.8) 704 (71.8) 187 (19.1) 71 (7.2)
Number of prior own egg embryo transfers		
0 1 2 >=3	20 (37.0) 19 (35.2) 9 (16.7) 6 (11.1)	321 (32.8) 422 (43.1) 134 (13.7) 103 (10.5)
Number of prior donor egg embryo transfers		
0 1 2 >=3	48 (88.9) 5 (9.3) 0 (0) 1 (1.9)	951 (97.0) 14 (1.4) 10 (1.0) 5 (0.5)

Continuous data is presented as mean ± standard deviation (SD), categorical data is presented as number (percentage). FET = frozen embryo transfer. BMI = body mass index

**Table 2:** FET cycle specific characteristics

Variable	Fluid resolved (N = 54)	No fluid (N = 980)
FET protocol		
Medicated True natural cycle	44 (81.5) 10 (18.5)	491 (50.1) 489 (49.9)
Endometrial thickness (mm)	8.8 (1.7)	9.9 (2.2)
Number of embryos transferred		
1 2 >=3	49 (90.7) 5 (9.3) 0 (0)	899 (91.7) 77 (7.9) 4 (0.4)
Good or best quality embryo(s) pre-freeze	53/53 (100%)	948/964 (98.3%)
Donor egg FET	6 (11.1)	65 (6.6)
PGT-M	3 (5.6)	77 (7.9)

Continuous data is presented as mean ± standard deviation (SD), categorical data is presented as number (percentage). FET = frozen embryo transfer. PGT-M = pre-implantation genetic testing for monogenic/single gene disorders

**Table 3:** Primary and secondary outcomes comparing study group where fluid was present on initial ultrasound evaluation and subsequently resolved prior to FET, relative to cycles without endometrial cavity fluid

Outcome	Fluid resolved (N = 54)	No fluid (N = 984)	RR	aRR
Live birth	19 (35.2)	335 (34.2)	1.0 (0.8–1.2)	1.0 (0.7–1.5)
+BhCG	26 (48.2)	548 (55.9)	0.9 (0.7–1.1)	0.9 (0.7–1.2)
Clinical intrauterine	21 (38.9)	460 (46.9)	0.9 (0.7–1.1)	0.8 (0.6–1.2)
Miscarriage	2/26 (7.7)	109/548 (19.9)	0.9 (0.8-1.0)	DNC
Ectopic	0 (0)	19 (1.9)		

Values are number (percentage). All analyses performed using log binomial regression adjusted for age at the time of retrieval, body mass index, number of prior pregnancies, diagnosis of PCOS, other ovulatory disorder, tubal factor, endometriosis, use of donor oocytes and number of embryos transferred. RR = risk ratio; aRR = adjusted risk ratio; CI = confidence interval; hCG = human chorionic gonadotropin; DNC = did not converge

## Discussion

In our study, live birth rates were similar between patients with resolved ECF compared to those in which ECF was never observed. Baseline patient characteristics between the two groups were grossly similar, with a few notable exceptions. We found that rates of tubal factor infertility were similar between the ECF and control group (11.1% vs. 10.6%, respectively), but rates of PCOS were higher in the ECF group (20.4 vs. 10.2%). In addition, we found that a disproportionately higher number of patients in the ECF group enrolled in a programmed hormonal replacement protocol rather than a true natural cycle protocol (81.5% vs. 19.5%, respectively). Lastly, we also found that patients who developed ECF had a thinner endometrium on average compared to those without ECF (8.8 mm vs. 9.9 mm, respectively).

Current literature pertaining to IVF outcomes in the context of transient, resolved ECF is sparse. We present the largest cohort of patients in whom ECF was detected and spontaneously resolved by embryo transfer. A comparable study by Akman et al. [1] evaluating implantation rates in fresh cycles among patients with resolved ECF compared to those without ECF found there to be similar implantation rates among patients with PCOS, but lower rates in those with tubal factor. This study did not examine live birth rates, was limited by excluding all other fertility diagnoses, and presented fewer cases of transient ECF overall (24 in PCOS patients, 14 with tubal factor). A study by Chien et al. [4] presented 20 fresh cycles with transient ECF accumulation of which only two conceived (10%), however these findings were compared to those with persistent fluid (of whom none conceived) rather than those without ECF, and its results did not reach statistical significance. The remainder of available studies examining the impact of ECF on IVF outcomes, including a recent meta-analysis by Liu et al. [13], are difficult to interpret in the context of our findings given ECF detected among patients was either persistent or its status was unknown at time of embryo transfer [8, 12].

While there are well documented reports of implantation failure with ECF in-situ [9, 13], our study and some existing reports suggest that transient ECF is a separate entity and may not bear the same detrimental effects [1, 12]. While some postulate that ECF may be embryotoxic and have lasting effects [1, 5, 11, 16], our findings appear to oppose this theory. It appears the threat of ECF is highest when persistent, thus the mechanism by which it inhibits implantation may be mechanical. This has been supported by several studies owing the detrimental effect of ECF to its magnitude and volume [2, 4]. A study by He et al. [9] found that clinical pregnancy rates among ECF patients with an anterior-posterior diameter (APD) between 1.0 and 3.4 mm was 35.5%, compared to 0% in patients with an APD of > 3.5 mm – notably in this study, patients with an APD > 3.5 mm were more likely to have persistent fluid rather than transient. The benign nature of transient fluid is also supported by existing studies observing self-resolving ECF after HCG administration, all of which concluded that there was no impact on clinical pregnancy rates compared to those without ECF [1, 12]. Our patients were not administered HCG, however the fact there was still no impact on live birth rates in our study among FET patients is noteworthy and reassuring.

In regards to etiology, hydrosalpinges and tubal factor infertility are often associated with the development of ECF in current literature [4, 9, 13]. However, the meta-analysis by Liu et al. [13] found that rates of tubal factor infertility were similar between ECF and non-ECF patients when hydrosalpinx was excluded. In our study, the rate of tubal factor infertility in the ECF group (11.1%) was similar to that of controls without ECF (10.6%). As mentioned previously, most patients in current literature with transient fluid were those with HCG stimulation at oocyte retrieval [1, 12], although this condition did not apply to our patients undergoing FET. In our study, patients with male factor infertility, endometriosis, unexplained infertility, and uterine factors in addition to tubal factor were all capable of developing transient ECF. Thus, we speculate the etiology of ECF in our study was heterogenous and not borne exclusively from hydrosalpinx or tubal factor.

It is important to note that 3 of the 5 patients excluded from our study who elected to proceed with embryo transfer despite persistent ECF were able to conceive, with one patient achieving live birth. One patient likely developed ECF secondary to hydrosalpinx at time of embryo transfer and conceived, but ultimately ended in miscarriage. While our study was not designed to examine live birth rates among patients with persistent ECF, clinical pregnancy and live birth was certainly still possible. This is consistent with the literature describing the possibility of pregnancy with persistent ECF. Most of the literature describing ECF as detrimental are in the context of hydrosalpinx, however as mentioned previously the etiology of ECF in our cohort is likely to have been diverse. It is not currently known if organic causes of ECF (such as hydrosalpinx or isthmocele) infer worse prognosis compared to those without gynecologic disease. Indeed, the chances of live birth after embryo transfer with functional ECF in-situ in the absence of organic disease has yet to be delineated and deserves further study to explain why some but not all patients with persistent ECF are able to conceive.

We also found that the number of patients who developed transient ECF enrolled in programmed cycles was disproportionately higher than those in true natural cycles (81.5% vs. 19.5%, respectively). This distribution stands in contrast to patients without ECF, who were almost equally split between protocols (50.1% programmed and 49.9% natural). Possible reasons for this include a higher proportion of PCOS patients in the ECF group than controls (20.4% vs. 10.2%, respectively), given irregular menses were among exclusion criterion for a natural cycle. In fact, the majority of ECF patients (39/44) who underwent a programmed cycle were primarily enrolled in this protocol. The remaining 5 initially planned for a true natural cycle but ultimately had inadequate mid-luteal serum progesterone level and ended up pursuing a programmed cycle. We also found that ECF patients had a thinner endometrium on average compared to those without ECF (8.8 mm vs. 9.9 mm, respectively). The reason as to why ECF patients were reported to have a thinner endometrium is speculative, however measurement inconsistencies due the presence of fluid, or a mechanical suppression effect on the endometrium may have been contributory. Lastly, given the majority of ECF patients underwent a programmed cycle, it is possible that supraphysiologic estrogen supplementation may have also played a role in the development of transient ECF [20]. It should be noted that the FET protocol employed the time of study in both natural and programmed cycles included embryo transfer 4 days after LH surge or exogenous progesterone start respectively, which is not line with current practice at the time of publication.

Compared with the limited number of existing reports concerning the prevalence and impact of ECF on ART outcomes, our study has several strengths. To our knowledge, ours is the first study in the literature examining the impact of transient ECF on live birth rates, as existing studies have been limited to implantation and clinical pregnancy rates. We also present the largest cohort of patients with ECF undergoing FET rather than fresh embryo transfer cycles. Furthermore, our data only includes the first FET performed per patient in an effort to reduce sampling error, as existing studies have used multiple cycles with ECF from the same patient due to its relatively low prevalence. Further, we were able to include a multivariate analysis adjusting for several independent variables to minimize confounding. Lastly, by using clinic linkage to a provincial outcome database, our results are not confounded by loss to follow-up.

Limitations of our study include its retrospective nature, given the possibility of missing or unreported information. Specifically, we were unable to determine the presence of Cesarean scar defect which may have been an organic cause of ECF that may have offered insight with respect to etiology and prognosis. Our patients had routine saline infusion sonohystogram (and some had laparoscopy) to rule out hydrosalpinx, although it is possible some patients had occult hydrosalpinges given hysterosalpingography (HSG) was not part of routine fertility evaluation. However, by including all patients with transient ECF regardless of etiology, our findings are more generalizable to the FET population with diverse infertility diagnoses who develop transient ECF. Secondly, given ECF is an uncommon entity, we saw significant disparity between the sample size of the study and control groups. In addition, while the use of both true natural and programmed protocols may have potential to introduce bias, the inclusion of both allows for greater generalizability of our findings among all FET patients and avoids producing a non-representative sample. Finally, the sample size of this study was powered to detect a 25% difference in live birth rates between the two groups without a significant difference observed. We recognize that a smaller difference in live birth rates may still be clinically significant with respect to IVF outcomes. Thus, further research is required to elucidate a possible more nuanced yet clinically significant effect of ECF on live birth rates.

In conclusion, our study did not detect a significant difference in live birth rates in patients with transient fluid and those without ECF, however a small and potentially clinically significant difference may yet exist. Further research is required to determine the various etiologies of ECF and their respective influence on IVF outcomes.

## Data Availability

Canadian Assisted Reproductive Technologies Register (CARTR Plus) provided birth outcome data for IVF cycles. Information on how register to access this database can be found at https://www.bornontario.ca/en/data/cartr-plus.aspx.
